# The effects of cognitive-behavioural therapy on mood-related ruminative response style in depressed adolescents

**DOI:** 10.1186/1753-2000-2-3

**Published:** 2008-01-29

**Authors:** Paul O Wilkinson, Ian M Goodyer

**Affiliations:** 1 Section of Developmental Psychiatry, Department of Psychiatry, University of Cambridge, Cambridge, UK

## Abstract

**Background:**

A mood-related ruminative response style increases the risk of onset and persistence of depression. This preliminary study investigated whether, in depressed adolescents, cognitive-behaviour therapy reduces mood-related ruminative response style. Whether specific factors within the rumination scale were differentially affected by CBT is also reported.

**Methods:**

26 depressed adolescents were randomised to receiving serotonin-specific reuptake inhibitor antidepressants (SSRI) plus psychosocial treatment as usual or SSRI and psychosocial treatment as usual plus CBT. Ruminative response style and depressive symptoms were measured at baseline and after 30 weeks of treatment, with the Responses to Depression Questionnaire and Mood and Feelings Questionnaire.

**Results:**

There were significantly greater reductions in ruminations in the CBT group compared to the non-CBT group (*p *= .002). There was no significant difference in the reduction in self-reported depressive symptoms between the groups. Rumination was reduced to levels of never-depressed controls in adolescents who had recovered from depression and received CBT. There were greater falls in the CBT group in the more pathological 'brooding' factor of rumination.

**Conclusion:**

These findings suggest that adding CBT to SSRI medication in the presence of active clinical care causes a greater reduction in mood-related ruminative response style in depressed adolescents. This may reduce the risk of future relapse.

**Trial registration:**

Current Controlled Trials ISRCNT83809224.

## Background

### Mood-related response style

The degree to which a person, when dysphoric, focuses attention on his or her symptoms, and the 'potential causes, implications and consequences of these symptoms', is referred to as mood-related ruminative response style (MRRS) [1]. It has been hypothesized that this mood activated or 'hot' thinking style is a component of cognitive vulnerability for the onset of clinical depression. Four possible pathological mechanisms have been suggested [2]: inappropriately using negative thoughts and memories to understand current circumstances; ruminative thoughts interfering with and diminishing problem-solving; ruminative thoughts interfering with and restricting the use of adaptive behaviour; if people ruminate and talk a lot about these ruminations, other people may find this wearisome and so be less likely to provide social support.

The Response Style Questionnaire (RSQ) [3] was developed to measure MRRS. High scores, indicating greater MRRS, predict longer and more severe episodes of dysphoric mood [3-5] and onset of a major depressive episode [6] in adults. High rumination is also associated with increased future depressive symptoms [7,8] and depressive episodes [9] in children and adolescents. In the latter study [9], rumination was highly correlated with depressive symptoms, and rumination predicted a persistent as opposed to a remitting major depressive episode, but not a remitting episode compared to no episode, when depressive symptoms were controlled for [10]. Although studies have not directly compared the effects of rumination in different age groups, it appears that rumination has qualitatively similar adverse effects in adolescence and adulthood.

Ruminating is significantly associated with neuroticism [4,11], hopelessness [11], faulty attribution [11] and dysfunctional attitudes [11] in adults and neuroticism in adolescents [12]. High rumination amplifies the effects of actual:ideal discrepancy on depressive symptoms [13]. Rumination mediates the effects of the dysfunctional thinking styles of internal, stable & global negative attributions; dysfunctional assumptions; self-criticism; and neediness on onset of a depressive episode over the following 2 1/2 years [6].

Preliminary evidence for a process independent of depressive symptoms has been reported from the experimental induction of ruminating thoughts. A ruminative procedure (focusing on one's symptoms, emotions and oneself) has been shown to worsen dysphoric mood in depressed but not non-depressed adults [14]. An experimental study demonstrated that high levels of trait rumination predicted reduction in positive affect after a task designed to lead to failure, but only in the group that had been trained to think abstractly about the causes, implications and consequences of situations (ie ruminate); and not in the group that were trained to think about the concrete sensory details of events [15]. The implication of these findings is that MRRS is a key component in amplifying of dysphoric mood, particularly in individuals with high levels of self-devaluative ideation and a temperamental predisposition to emotional response to environmental cues.

In view of the evidence of the harmful effects of rumination, it is important to identify treatments that reduce levels of rumination in individuals who are depressed, or at risk of depression. This study will investigate whether specific treatment reduces rumination.

### Factor analysis of the response style questionnaire

As noted above, studies of ruminative thinking style have traditionally used the Ruminative Responses sub-scale of the RSQ [3]. It is possible that some items of the RSQ are proxy measures of depressive symptoms, which may confound the findings that high rumination predicts depressive symptoms. Factor analytic studies have been carried out on the RSQ. Five studies have recruited adult community samples [16-20]; two have recruited clinic samples of depressed adults [11,21]; one study recruited a community sample of adolescents [8]. Inspection of the factors across these studies showed that the same items normally loaded onto equivalent factors. In particular, a more pathological factor (often labeled 'brooding') has been found in most studies, which contained items that were unlikely to be influenced by levels of depressed mood; this factor was more strongly associated with current and/or future depressive symptoms than the other factors. This suggests that that it may be inappropriate and inefficient to treat the rumination subscale as unidimensional.

### Cognitive-behavioural therapy

In the UK, cognitive-behavioural therapy (CBT) is a psychotherapy recommended as a first-line treatment for mild to moderate depression in children and adolescents if routine psychosocial management is unsuccesful [22]. This treatment has multiple components, in particular it aims to correct negative thinking styles that are liable to be maladaptive, and in addition encourages positive behaviours as therapeutic distractors. Part of the treatment encourages taking part in positive activities rather than sitting alone, doing nothing, a time when negative and ruminative thoughts may occur, worsening mood. Theoretically, this treatment should therefore lead to a reduction in ruminative response styles.

Studies in adolescents have demonstrated that CBT is more effective than control treatments for adolescent depression. However, more recent meta-analyses [23] have shown smaller effect sizes than earlier ones [24,25], suggesting that it may not be as effective as previously thought. The largest CBT study to date in adolescents, the Treatment for Adolescents With Depression Study (TADS) [26] failed to show an advantage of CBT over placebo, although combined antidepressant plus CBT treatment was more effective than antidepressant alone at 12 weeks. There was no difference between active treatments at 36 weeks.

A randomized controlled trial carried out in routine English National Health Service clinics and comparing serotonin specific reuptake inhibitor antidepressant (SSRI) plus active psychosocial treatment as usual (TAU) against SSRI and TAU plus CBT for severely depressed adolescents demonstrated no advantage of adding CBT to treatment (Adolescent Depression Antidepressants and Psychotherapy Trial, ADAPT [27]). These were similar to the results of a similar study in the USA [28]. Lack of superiority in these two more recent trials may reflect a greater level of psychosocial intervention in the non-CBT group; possibly non-specific psychological support is as effective as the specific components of CBT. In all these trials the primary outcome measure was current depressive symptoms or levels of psychosocial impairment. The UK RCT provided an opportunity to test the effects of CBT on the thinking styles of depressed adolescents in this preliminary study in a subset of the ADAPT participants.

### Hypotheses

The primary hypothesis of this preliminary study was that adding CBT to SSRI plus psychosocial treatment as usual would cause greater reduction in rumination. If true then some support is provided for CBT having particular positive effects on depressogenic cognitions that neither SSRI nor active routine clinical care provide. A secondary hypothesis was that rumination levels would be no different to those of healthy controls among adolescents recovered from depression (less than two depression symptoms for at least 8 weeks [29]) who had received CBT. We further hypothesized that CBT would have specific effects on the ruminative factor of brooding but not on reflection (as identified by Burwell and Shirk in adolescents [8]), as brooding contains response styles that are likely to be more maladaptive and increase the risk of depression, and so more likely to be targeted by CBT. In addition, it is probably more important to target brooding as reducing brooding is more likely to reduce depressive symptoms, concurrently and in future.

## Methods

### Participants

#### Depressed cases

Consecutive referrals to community child and mental health clinics in Cambridge and Huntingdon who met eligibility criteria were invited to take part in the Adolescent Depression Antidepressants and Psychotherapy Trial (ADAPT) and this study, the Thinking Styles Study. There were fewer participants in this study than ADAPT, because only Cambridgeshire participants were recruited, and recruitment started later than the wider ADAPT study. Inclusion criteria for being in both studies were: ages 11–17 (inclusive); current DSM-IV major depressive disorder; and evidence of social, family or school impairment. Cases were excluded if they were too unwell for the treatment study (in practice, this referred to patients who required immediate antipsychotics or immediate admission – 2% of all depressed participants who were assessed in both centres; suicidal thoughts or acts were not exclusion criteria); had contraindications to SSRI use; had a significant learning disability; if an organic cause for depression, schizophrenia or bipolar disorder was present (as determined by K-SADS-PL interview); subjects and/or carers were unable to complete research questionnaires; a history of epilepsy or other major neurological disease; taking psychoactive drugs (prescribed or illicit), other than antidepressants, which would be active at time of interview (as elicited by K-SADS-PL interview and direct questioning on recent drug use).

#### Controls

Healthy controls were recruited to demonstrate whether rumination levels were similar to those of healthy controls after CBT, or remained high. Control subjects were recruited from two Cambridgeshire schools. Exclusion criteria were: current diagnosis of mental illness as defined in DSM-IV; lifetime history of major depressive disorder; current severe psychosocial stress; current neurological disorder; regularly taking psychoactive drugs (prescribed or illicit) which would be active at time of interview (as elicited by K-SADS-PL interview and direct questioning on recent drug use); learning difficulties necessitating special schooling; not speaking fluent English.

### Measures

The *Responses to Depression Questionnaire, RDQ*, is a slight modification of the original Response Styles Questionnaire (Nolen-Hoeksema & Morrow, 1991), with wording of some items slightly modified to make it more appropriate for adolescents [30]. It is a 39-item questionnaire asking participants what they habitually think, do or feel when they experience low mood (not clinical depression). There are 21 negatively worded rumination items. Past research demonstrates good evidence for discriminant validity and stability for the original questionnaire [3]. While stability has not been demonstrated for the adolescent version, predictive validity for future depressive disorder and depressive disorder has been demonstrated [9]. Each item was scored from 0 to 3. Scores were obtained by adding up the rumination items for total rumination score; and by adding up the scores for all items within each factor. 5 out of 6 items labeled as brooding (not Think, "why do I have problems others don't?"), and all 4 items labeled reflection, in the Burwell and Shirk's study [8], are present in the original version of the RDQ, as used in this manuscript.

The *Kiddie – Schedule for Affective Disorders and Schizophrenia – Present and Lifetime Version (K-SADS-PL) *is a semi-structured interview, which provides DSM-IV diagnoses, with reliability proven in past research [31]. All screen sections were applied, and if screening was positive, the full section was administered. In this study, agreement between two independent raters was 100% for the diagnosis of major depression.

The *Mood and Feelings Questionnaire, MFQ *[32] is a 33 item self-rated questionnaire of depressive symptoms and was completed by all participants. It has good test-retest reliability (ICC = 0.8, [33]; Pearson's *r *= 0.78, [34]). A cut-off of 28/29 has been shown to optimally discriminate adolescents with major depression from those with sub-threshold depression or no depressive disorder [33].

The *Raven's Standard Progressive Matrices, SPM*, [35] was designed as a test of eductive intelligence, the non-verbal ability to identify, generate and manipulate new concepts. It can be thought of as being related to performance IQ. Eductive intelligence may affect ability of individuals to utilise CBT successfully, and so between-group differences may confound the results. There are 60 separate patterns or sets of patterns, each with one piece missing; and six or eight possible answers for each pattern, only one of which is correct. The test was explicitly not timed, so that final scores would reflect subjects' eductive abilities and not be influenced by time pressures or processing speed. Raven's SPM scores were transformed to age-scaled score bands (higher bands represent higher intelligence; bands run from 1 to 8; bands 4 and 5 are, respectively, the bands below and above the 50^th ^centile). Where participants' ages were above the maximum ages for the oldest age bands (up to 15 years, 8 months), they were scored as if in the oldest age band.

### Test procedure

After consent was obtained, participants were randomised to a 28 week course of SSRI and active psychosocial treatment as usual plus CBT (CBT group) or SSRI and active psychosocial treatment as usual without CBT (TAU group) by a 1:1 treatment allocation ratio by remote computer. TAU was conducted in an empathic and reflective framework with monitoring of mental state, psychoeducation, parental support, active listening, and liaison with other agencies. Co-morbid diagnoses were made and discussed with families; in the case of anxiety disorders, it was stated that antidepressants should help to reduce anxiety, both directly and through reducing depressive symptoms. Additional interventions during sessions in those randomized to CBT were: engagement and goal setting, emotional recognition, self-monitoring, self-reinforcement and activity scheduling, challenging negative thinking and cognitive restructuring, social problem solving, and communication skills; where required, establishing hierarchies, exposure and reward techniques were included; homework tasks were given for between sessions. These CBT strategies are similar to those used in TADS [26]. Such specific CBT strategies, including education about thinking and using distraction instead of ruminating, were explicitly forbidden in the TAU group, with the exception that graded exposure (without asking about or dealing with cognitions) was allowed for adolescents not attending school. Similarly to TADS [26] (but differently to some CBT studies), which components of the manual were chosen for each session was flexible and tailored to the needs and developmental abilities of the adolescent, rather than following a fixed order across the treatment course.

All medication monitoring, basic psychosocial treatment as usual and CBT was provided by the first author. Neither the patients nor this investigator were blinded to treatment. All treatment sessions were audiotaped and a random selection of study sessions were listened to by members of the ADAPT team in Manchester, to assure quality and model-fidelity of CBT, using the cognitive therapy scale [36]; the scale mid-point (39) was deemed to represent acceptable CBT quality. Other specific interventions, such as family therapy, could be given. These were kept to a minimum in the first 12 weeks after randomisation. After 12 weeks (the primary outcome timepoint for ADAPT), cases who were still depressed had treatment reviewed; in some cases CBT was able to be given to the non-CBT group after 12 weeks, if the clinician and family deemed it the most appropriate treatment and if it was available. This was because we did not feel it ethical to deny the treatment that we hypothesized was the more effective treatment for the full 28 weeks of the treatment study. More detailed description of ADAPT treatment is provided in the primary clinical outcome paper [27].

Depressed participants were interviewed by the author at baseline, and at one follow-up session, which was planned to be 30 weeks after baseline. All the above measures were used at baseline testing. RDQ and MFQ were applied at the follow-up interview. Raven's SPM was only included at baseline to check if groups were balanced, and were not tested at follow-up. Interviewing took place in clinic. Controls were interviewed by the first author on one occasion in their school or clinic, using K-SADS-PL, RDQ and MFQ.

Research was carried out according to the principles of the Helsinki declaration. Ethical approval was obtained from the Cambridge (01/257) and Huntingdon (H2/765) Local Research Ethics Committees. Written informed consent was obtained from each subject and one of their parents.

### Statistical analysis

Analysis of covariance (ANCOVA) was used to test whether follow-up scores in the primary outcome measure, RDQ rumination, was significantly different in the CBT and TAU groups, controlling for baseline scores. ANCOVA was also used to test if final MFQ differed between the two groups, controlling for baseline MFQ. As secondary analyses, follow-up scores in adolescents recovered from depression [less than two symptoms of depression for the previous 8 weeks [29]] were compared between each of the treatment groups and controls; and follow-up scores in the rumination factors were compared between CBT and TAU groups. Primary analysis was by intention-to-treat (such that participants were analysed in the group they were randomized to), subject to availability of data. This minimizes bias in that it increases the chance that groups are balanced at baseline, as reasons for changing group are non-random (in this cases, cases less responsive to treatment are more likely to be moved to the CBT group, reducing the effect size). This method has problems in a process study where one of the questions is 'how does a specific treatment actually change a variable'. Therefore per protocol analysis (where only participants remaining in the group they were randomized to) was also performed.

Baseline characteristics between groups were compared by Student's *t *test when data were normally distributed, by Mann-Whitney test when not normally distributed, and by Fisher's exact test for categorical variables.

A threshold *p *value of 0.05 was deemed statistically significant. Data analysis was carried out by SPSS version 12.0.1 for PC.

## Results

### Baseline sample characteristics

33 participants from the Cambridgeshire arm of the treatment study were eligible to take part in, and were assessed during the time-frame of, the thinking styles study; of these, 26 (78%) consented to take part. 11 were randomized to SSRI and TAU, 15 were randomized to SSRI plus CBT. 23 participants consented to take part in the final follow-up assessment, 10 from the TAU group, 13 from the CBT group. Mean (standard deviation) for cognitive therapy scale ratings for CBT sessions was 55.1 (10.4). Only one session was below 39 (probably because in-patient treatment needed discussing in that session). There were no significant differences in age, gender, depression severity nor baseline outcome variables between the three who dropped out and those who remained in the study (all *p *> .2).

Baseline data on demographics, depression severity, rumination and time between assessments of the participants who remained in the study are presented in table 1. Depression severity was higher than that of the whole ADAPT sample [ADAPT mean (sd) MFQ, 37.9 (11.9); *t*(df22) = 2.91, *p *= 0.01]. There was no significant difference in rumination (p = .9) between the two depressed groups. There were no significant differences between depressed groups (p > .6) and between depressed and control groups (p > .1) on age, gender and Raven's scores. Both depressed groups had significantly higher baseline RDQ rumination and self-rated depressive symptoms than the control group (all *p *< .001).

**Table 1:** Baseline characteristics of groups

	TAU (n = 10)	CBT (n = 13)	Controls (n = 38)
Sex, male:female	4:6	3:10	11:27
Age	15.4 (1.1)	15.2 (1.1)	14.8 (1.0)
RSPM band	4.3 (1.7)	4.2 (1.6)	4.6 (1.7)
MFQ	42.5 (10.1)	43.8 (8.1)	5.1 (4.2)
RDQ rumination	30.4 (13.1)	30.1 (9.6)	8.37 (8.7)
Time between assessments in weeks, median (range)	34 (27–42)	32 (28–61)	-

*Note: *Unless otherwise stated, continuous measures are presented as mean (standard deviation)

TAU = group allocated to SSRI and treatment as usual; CBT = group allocated to SSRI and CBT

RSPM = Raven's Standard Progressive Matrices; MFQ = Mood and Feelings Questionnaire, higher scores represent more depressive symptoms; RDQ = Responses to Depression Questionnaire, higher scores represent higher rumination.

### Treatment received

The median interval between assessments was 32 weeks. During the 28 weeks of the treatment study, participants in the TAU group had a mean of 11.7 (sd 2.7) treatment sessions and those in the CBT group had a mean of 13.6 (sd 5.4) treatment sessions (*t*(df = 21) = 1.02, *p *= .3). 2 participants allocated to TAU received CBT during the treatment study. 1 participant in the CBT group was admitted to a psychiatric unit. 2 participants in the CBT group and 1 in the TAU group received out-patient family therapy.

### Outcome measures at endpoint

#### Primary measures (intention to treat analysis)

**Figure 1:**
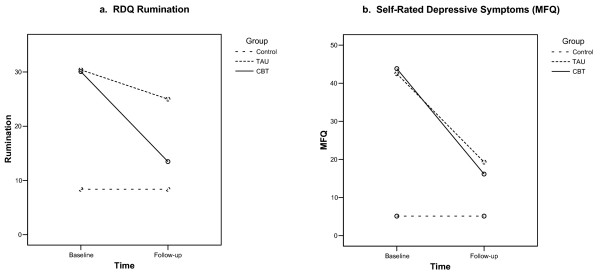
**Primary outcome measures at baseline and follow-up for depressed groups and controls**. *Note: *Controls were only measured at baseline. A straight line at this baseline value was drawn for illustrative purposes. TAU = group allocated to SSRI and treatment as usual; CBT = group allocated to SSRI and CBT. RDQ = Responses to Depression Questionnaire; MFQ = Mood and Feelings Questionnaire.

Figure 1 shows the scores on the primary outcome variables and MFQ in the two depressed groups at both time points, together with the control group single score. Mean (standard deviation) scores at follow-up for the TAU and CBT groups respectively were: ruminations: 25.0 (14.7) and 13.5 (9.5); MFQ: 19.3 (23.8) and 16.2 (14.3).

Controlling for baseline scores, ruminations were significantly lower at follow-up in the CBT compared with the TAU group (*F*(df 1,20) = 12.0, *p *= .002). Secondary per protocol analysis also demonstrated significantly lower rumination in the CBT group than those in the TAU group who did not receive CBT (*F*(df 1,18) = 16.6, *p *= 0.01). Secondary per protocol analysis including the 16 who did not receive non-allocated CBT, family therapy nor in-patient admission demonstrated significantly lower rumination in the CBT group (*F*(df 1,14) = 24.2, *p *< 0.001).

There was no significant difference in final MFQ between the two groups, controlling for baseline scores (*F*(df 1,20) = 0.1, *p *= 0.7). In addition, there was no significant difference between groups in the MFQ or other outcome measures (including clinical, social function and quality of life variables) in separate assessments carried out by a research assistant blinded to treatment allocation at 0, 6, 12 or 28 weeks during the treatment study within this sample (details available from first author) and there were no significant between-group differences over the whole treatment study with 208 participants [27].

#### RDQ measures in recovery

7 participants in each treatment group met pre-determined criteria for recovery. Table 2 shows scores in recovered patients and controls. In both groups, final MFQ was similar to that of never-depressed controls. Rumination remained higher in those who had not received CBT (effect size = 1.9, *p *= 0.023). However those who had received CBT now had rumination levels very similar to controls (effect size = 0.18, *p *= 0.5).

**Table 2:** Final depressive symptoms and response styles in participants recovered from depression and healthy controls

	Controls (*n *= 38)	Recovered, TAU (*n *= 7)				Recovered, CBT (*n *= 7)			
		
			TAU vs controls				CBT vs controls		
		
		Final	Cohen's *d*	*Z*	*p*	Final	Cohen's *d*	*Z*	*p*
MFQ	5.1 (4.2)	4.9 (4.0)	-0.05	0.1	0.9	6.0 (4.7)	0.21	0.2	0.8
Rumination	8.4 (8.7)	26.4 (13.2)	1.9	2.3	0.023	9.9 (7.2)	0.18	0.7	0.5

*Note: *Continuous measures are presented as mean (standard deviation)

TAU = group allocated to SSRI and treatment as usual; CBT = group allocated to SSRI and CBT

MFQ = Mood and Feelings Questionnaire

Cohen's d = effect size (difference in means/common standard deviation)

#### RDQ factors

Table 3 shows mean (sd) baseline and final scores, together with ANCOVAs of between-group differences in final scores, controlling for baseline scores.

**Table 3:** Baseline and follow-up scores for rumination factors

	TAU		CBT		Controls	*F *(df1,20)	*p*
*Number of subjects*	10		13		38		

	Baseline	Final	Baseline	Final			

Rumination							
Brooding	8.2 (3.6)	6.1 (4.0)	9.5 (2.5)	3.5 (2.6)	2.6 (3.1)	11.5	.008
Reflecting	4.1 (3.2)	4.1 (2.5)	2.8 (2.7)	2.5 (2.8)	1.4 (2.0)	1.1	.3

*Note: *Continuous measures are presented as mean (standard deviation).

TAU = group allocated to SSRI and treatment as usual; CBT = group allocated to SSRI and CBT

Post-treatment brooding was significantly lower in the CBT group, controlling for baseline scores. There was no post-treatment difference in reflectiveness.

## Discussion

Findings from this preliminary study suggest that cognitive-behavioural therapy together with antidepressant medication and TAU result in a significantly greater reduction in total rumination in depressed adolescents than medication+TAU.

As hypothesized, the rumination factor labeled 'brooding' by Burwell and Shirk [8], which is associated with greater future depressive symptoms, improved significantly more among those given CBT. There was no significant effect of CBT on 'reflection', which was not found to be associated with future depressive symptoms by Burwell and Shirk. This suggests that CBT is better at reducing the symptoms of rumination that are more likely to lead to future depressive symptoms.

Schmalling and colleagues [37] failed to demonstrate that problem-solving therapy was more effective than antidepressants at reducing rumination in a randomized controlled study. The authors commented that a therapy that cultivated a different way of thinking (such as CBT), rather than teaching the episodic skill of problem-solving, may be needed to reduce impairment. While problem solving is one component of CBT, possibly extra components of CBT (such as cognitive restructuring and encouraging distraction rather than rumination) are needed to reduce rumination. Watkins and colleagues [38] and Kingston and colleagues [39] demonstrated that (respectively) rumination-focused or mindfulness-based CBT reduced depressive symptoms and rumination in adults in partial remission from depression. However, in both cases, reductions in rumination may have been secondary to the greater reductions in depressive symptoms in CBT groups. Jain and colleagues [40], in a sample of students reporting 'distress', demonstrated that training in mindfulness meditation led to greater falls in rumination but no there was no post-intervention difference in distress levels compared with a relaxation training control group; and that rumination partially mediated mindfulness meditation's effects on reducing distress.

In this study, there was random allocation to treatment and the groups did not significantly differ on baseline severity and demographic variables; therefore we can conclude that CBT is likely to responsible for this reduction in depressogenic cognitive ruminations in depressed patients. There was no significant difference in the change in depressive symptoms between the two groups. This suggests that CBT either itself directly reduces rumination, or affects a third factor that reduces rumination, rather than the cognitive changes arising as a consequence of a non-specific improvement in overall depressive symptoms. Thus CBT does appear to improve response style in depressed adolescents even though it does not contribute to the overall improvement in short term outcome [27].

Rumination is higher in adults recovered from depression than never-depressed controls [17]. Residual depressive symptoms following remission increase the risk of subsequent relapse [41]. This study provides preliminary evidence that CBT (but not TAU) leads to a reduction in rumination to levels seen in healthy controls at recovery (although a larger sample is needed to confirm no true difference). As rumination predicts onset of depression, it is possible that CBT given during partial remission will reduce the risk of relapse of adolescent depression, as has been demonstrated for depressed adults [42,43]. This reduction in relapse may be mediated by an improvement in dysfunctional mood-related ruminative response style. Longer follow up of treated adolescents is needed; in particular it would be useful to investigate if giving CBT in partial remission increases the chance of full remission and/or reduces the risk of future rumination and relapse. It may be useful for future studies to test whether it is more cost-effective to start using CBT in partial remission or as a 1^st ^line treatment.

### Limitations

This preliminary study was limited by the small number of participants, limiting power. This could have led to type 2 errors. While this would not lead to a falsely low p value for the primary findings, it does mean that the study can be less confident that there are no significant differences between potential confounding variables. In particular 40% of the TAU group but 23% of the CBT group were male, a non-statistically significant finding at this small *n*. However, there were similar treatment effects, with CBT being superior, within the two genders [males *F*(df 1,4) = 13.1, *p *= 0.02; females *F*(df 1,13) = 3.4, *p *= 0.087]. Therefore results were more likely to be have been due to CBT than ruminations falling by more in females. Likewise, we cannot state with confidence that there is no difference in effect on MFQ between treatments. However, the effect size (difference in means/standard deviation) is very small, at 0.17 (compared with 0.98 for rumination). A sample size of over 500 in each arm would be needed to have 80% power of detecting such a difference to be significant at alpha threshold of 0.05. So we can say that even if an effect of CBT on depressive symptoms accounts for some of the improvements in rumination, this is only a very small effect, and most of the effect of CBT on rumination is through another mechanism. We also cannot state with confidence that rumination levels are not different to those of healthy controls after CBT, but again effect size is very small (0.18).

Neither the participants nor research assessor were blind to treatment group, which may have led to information bias. There is likely to be less bias with a self-report questionnaire, as used, than an observer-rated interview. However, participants may have recognized that questions on ruminative responses were about areas that CBT should have improved and therefore given more positive answers. The questionnaire tries to reduce this potential bias by asking participants to 'Please tick what you **generally **do, not what you think you should do'.

Two participants in the non-CBT group did receive CBT, due to it being thought to be ethically inappropriate to not provide CBT for 30 weeks. This may have reduced between-group differences. However per-protocol analyses, excluding those who received CBT they were not allocated to, and excluding all those who received extra treatments, had similar effects to the main intention-to-treat analysis.

While this study demonstrated a greater fall in rumination in those receiving CBT, it did not demonstrate a greater reduction in depressive symptoms. Therefore it is not possible to draw a definitive conclusion that this fall in rumination will lead to any clinical benefit. Instead, future studies need to be longer and measure rumination and depressive symptoms at multiple time points; they should investigate whether a greater fall in rumination mediates later reductions in depressive symptoms or relapse rates.

This study was not able to demonstrate which component of CBT was responsible for reducing rumination. We would hypothesise that it is the encouragement to be more active rather than sitting at home thinking about problems rather than restructuring the content of thoughts about the self to be more positive. A study randomizing participants to two different types of CBT is needed to test this. Some preliminary evidence is provided by one study of depressed adults [44], which demonstrated that behavioural activation therapy (which encouraged patients to be more active and not ruminate) was more effective at treating depression than more traditional CBT (with more of an emphasis on cognitive restructuring). This led the authors to conclude that treatment should address the *behaviour *of thinking, rather than the *content *of thinking.

With the two major limitations of the small sample size and the fact that the CBT therapist administered outcome questionnaires, any conclusions can only be tentative until findings are replicated in a larger study with better blinding.

### Clinical implications

This preliminary study has suggested that adding CBT to antidepressant medication may reduce mood related ruminative thinking style independently of an effect on depressive symptoms. This may reduce the cognitive risk of future episodes. There may therefore be an important role for using CBT for patients in partial remission from depression, which needs testing in further studies.

## Abbreviations

ADAPT: Adolescent Depression Antidepressants and Psychotherapy Trial; CBT: cognitive behavioural therapy; K-SADS: Kiddie – Schedule for Affective Disorders and Schizophrenia – Present and Lifetime Version; MFQ: Mood and Feelings Questionnaire; SPM: [Raven's] Standard Progressive Matrices; RCT: randomized controlled trial; RDQ: Responses to Depression Questionnaire; SSRI: serotonin specific reuptake inhibitor antidepressant

## Competing interests

The author(s) declare that they have no competing interests.

## Authors' contributions

PW and IG designed the study, analysed the data and wrote the manuscript. PW collected data. Both authors read and approved the final manuscript.
